# Temporal Histological Tissue Responses Following Fractional Nonablative 1470 nm Laser Exposure: A Pilot In Vivo Human Study

**DOI:** 10.1093/asjof/ojag161

**Published:** 2026-09-02

**Authors:** Paweł Kubik, Wojciech Gruszczyński, Aleksandra Pawłowska, Maciej Malinowski, Maria-Luiza Piesiaków, Brygida Baran, Agnieszka Pawłowska-Kubik, Łukasz Kodłubański, Dariusz Grzanka, Paulina Antosik, Bartłomiej Łukasik

## Abstract

Fractional nonablative lasers enable controlled dermal heating while preserving epidermal integrity. The 1470 nm wavelength shows strong water absorption and moderate penetration, but in vivo human histological data on early tissue responses remain limited. The aim of this report was to characterize early, time-dependent histological responses and assess short-term histologic tolerability of a fractional 1470 nm laser (LaserMe). Eighteen healthy adults were enrolled and allocated to 6 treatment groups according to energy setting and spot spacing. For the present analysis, only samples treated at 28 and 50 mJ/point were included, because the 5 mJ/point setting produced no measurable histological response. Clinical assessment and full-thickness skin biopsies were performed at baseline, immediately after treatment, and on Days 3 and 10. Histology was evaluated using hematoxylin–eosin and Masson's trichrome staining. Treatment produced spatially confined micro-injury zones with the preservation of surrounding tissue. Immediately posttreatment, localized changes were observed at the dermal–epidermal junction with minimal epidermal involvement. By Day 3, re-epithelialization and basement membrane restoration were evident. By Day 10, epidermal continuity was fully restored, with reduced dermal thermal alteration and mild inflammatory infiltrates consistent with reparative processes. Higher energy increased dermal penetration without extending superficial epidermal damage. Fractional 1470 nm laser exposure induces a predictable, localized histological response consistent with possible early tissue remodeling and suggests favorable short-term histologic tolerability in healthy adult skin. This exploratory preliminary report provides mechanistic insight into nonablative laser–tissue interactions and was not designed to assess clinical efficacy or long-term safety.

**Level of Evidence:** 4 (Therapeutic) 
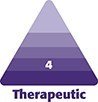

Over the past several decades, advances in laser technology have profoundly transformed aesthetic and dermatologic practice. Early laser-based interventions were predominantly ablative and relied on extensive tissue vaporization to induce skin resurfacing and subsequent tissue regeneration.^1,2^ Although clinically effective, these approaches were associated with prolonged recovery times, increased risk of complications, and limited applicability across diverse patient populations. Consequently, both technological development and clinical interest have progressively shifted toward modalities that promote controlled tissue stimulation while preserving epidermal integrity and minimizing adverse effects.^3^ A major milestone in this transition was the introduction of fractional photothermolysis in 2004.^4^ By delivering laser energy in a spatially fractionated pattern, fractional systems generate microscopic columns of thermal injury “microthermal zones,” surrounded by intact tissue. This preserved tissue serves as a reservoir for rapid keratinocyte migration and dermal repair, enabling effective tissue remodeling with reduced downtime and an improved safety profile.^4,5^ Since its introduction, fractional technology has become a cornerstone of contemporary laser dermatology, with broad applications including skin rejuvenation, scar revision, pigmentary disorders, and photodamage.^3^

The biological response to fractional laser exposure is governed by several interdependent parameters, including wavelength, pulse duration, energy density, and spatial distribution of microthermal zones.^6^ Among these factors, wavelength selection plays a central role, because it determines the dominant chromophore, penetration depth, and confinement of thermal effects within the skin.^7,8^ Water, the primary chromophore in cutaneous tissue, exhibits wavelength-dependent absorption characteristics that critically influence the extent and localization of laser-induced heating.^9^

Within this context, the 1470 nm wavelength occupies a distinctive position among nonablative mid-infrared laser systems. Because of its strong absorption by water and moderate penetration depth, radiation at 1470 nm enables selective photothermal interaction within the dermis while largely sparing the epidermis. This absorption profile allows precise heating of collagen-rich tissue compartments, promoting extracellular matrix remodeling processes and features associated with collagen reorganization, without inducing surface ablation. When delivered in a fractional pattern, 1470 nm laser energy creates evenly distributed microthermal zones that are intended to stimulate controlled dermal responses with minimal disruption of the epidermal barrier.^10^

In recent years, clinical studies have demonstrated that nonablative fractional laser systems operating at 1470 nm are effective and well tolerated in the treatment of various dermatologic conditions, including acne scars and striae distensae, with a favorable safety profile and limited downtime.^10-14^ These studies have confirmed clinical efficacy and patient-reported improvement across multiple indications. However, available evidence is predominantly outcome-oriented and focuses primarily on clinical endpoints.

In contrast, detailed in vivo histological data characterizing the depth, morphology, and temporal evolution of tissue responses induced by fractional 1470 nm laser exposure in human skin remain limited. In particular, systematic analyses correlating delivered energy parameters with microscopic patterns of epidermal and dermal interaction and their subsequent regenerative dynamics over time are scarce. Such data are essential for optimizing treatment protocols, defining safety margins, and advancing the mechanistic understanding of nonablative fractional photothermal stimulation.

The LaserMe system is a fractional, nonablative diode laser platform operating at a wavelength of 1470 nm and delivering energy in multi-millisecond pulses under contact-mode conditions. By modulating exposure time while maintaining constant beam power and spot size, the system enables precise control over delivered energy and spatial distribution of microthermal zones. This configuration provides a suitable model for investigating parameter-dependent tissue responses in human skin.^12,15,16^

The present study was therefore designed to evaluate the safety profile and histological effects of fractional 1470 nm laser exposure using the LaserMe device in an in vivo human model. By combining macroscopic clinical assessment with detailed histopathological analysis at defined time points following treatment, this investigation aims to characterize both immediate and time-dependent tissue responses induced by different energy settings, thereby contributing to a more comprehensive understanding of mechanistic aspects of nonablative fractional laser–tissue interaction.

## METHODS

### Study Population

Eighteen healthy adult volunteers (7 women and 11 men; age range: 35-68 years; mean age: 46.8 years) were enrolled in this prospective exploratory study. All participants provided written informed consent before inclusion. Eligibility was confirmed through medical history review and physical examination. Only individuals in good general health, without dermatologic or systemic conditions that could interfere with wound healing, were included. Participants were allocated to 6 treatment groups according to energy level and spot spacing, as detailed in Table 1. For the purposes of the present histological analysis, only groups treated at 28 and 50 mJ/point (Groups II, III, V, and VI) were included, as the 5 mJ/point setting (Groups I and IV) did not produce measurable histological changes. These analyzed groups differed in both delivered energy (28 vs 50 mJ/point) and spacing between laser exposure points (1.0 vs 4.0 mm).

**Table 1.** Study Group Allocation, Treatment Parameters, and Case Identification

Group	Case IDs (*n* = 3)	Energy (mJ/point)	Exposure time (ms)	Spacing (mm)	Anatomic sites
I	1LH, 2LH, 3LH	5	3.0	4.0	Abdomen, back, leg
II	1MH, 2MH, 3MH	28	16.8	4.0	Back, back, back
III	1HH, 2HH, 3HH	50	30.0	4.0	Back, back, back
IV	1LL, 2LL, 3LL	5	3.0	1.0	Abdomen, abdomen, leg
V	1ML, 2ML, 3ML	28	16.8	1.0	Back, abdomen, back
VI	1HL, 2HL, 3HL	50	30.0	1.0	Back, back, back

Case ID nomenclature: number = participant, first letter = energy level (L = low/5 mJ, M = medium/28 mJ, H = high/50 mJ), second letter = spacing (H = high/4.0 mm, L = low/1.0 mm). Groups II, III, V, and VI are included in the present manuscript. Groups I and IV (5 mJ/point) produced no measurable histological response. Beam power constant at 2 W across all groups; energy modulated through exposure time. Maximum diode source output: 2 W. All participants were healthy adults aged 35 to 68 years (18 total: 7 women, 11 men; mean age 46.8 years).

### Exclusion Criteria

Exclusion criteria included: use of systemic or topical retinoids within 6 months before study enrollment; excessive tanning or recent ultraviolet exposure; active dermatologic or connective tissue diseases associated with photosensitivity (including systemic lupus erythematosus, collagenopathies, and cutaneous porphyria); active herpes simplex infection; use of photoreactive medications or cosmetics (including tetracyclines, corticosteroids, immunosuppressive agents, anticoagulants such as dipyridamole or coumarin derivatives, cosmetics containing thyme extract, or herbal products containing *Hypericum perforatum*); immunodeficiency disorders; uncontrolled diabetes mellitus; acquired vitiligo or disorders of melanin synthesis; tattoos within the treatment area; regular use of nonsteroidal anti-inflammatory drugs; and previous cosmetic or aesthetic procedures in the treatment area (assessed on a case-by-case basis by the investigator).

The study was conducted in accordance with the principles of the Declaration of Helsinki and the requirements of ISO 14155:2020 (clinical investigation of medical devices for human participants Good Clinical Practice). The study protocol was approved by the local Ethics Committee of the Medical Chamber in Gdańsk, Poland (approval no. KB-5/93/2022; October 25, 2022).

### Laser Device

The medical device used in this study was the LaserMe diode laser system emitting at a wavelength of 1470 nm (Berger & Kraft Medical Sp. z o.o., Poland). The system operates in fractional, nonablative contact mode. Laser energy is delivered as multi-millisecond pulses with constant beam power and adjustable energy density ranging from 5 to 50 mJ/point. The spacing between laser exposure points is selectable between 1.0 and 4.0 mm. The maximum output power of the diode source is 2 W.

### Treatment Protocol

Laser treatment was performed using either a medium energy density of 28 mJ/point or the maximum tested energy density of 50 mJ/point, both with a laser point spacing of 1.0 or 4.0 mm. Energy per point was controlled by modulating exposure time while maintaining constant beam power (2 W): 16.8 ms for 28 mJ/point and 30 ms for 50 mJ/point. A single pass was performed per treatment area with no overlap between laser exposure points. Participants selected the treatment site from predefined anatomical regions (back, abdomen, or limbs) to ensure comparable skin thickness and histological characteristics across samples. All procedures were performed under standardized conditions.

### Study Procedures and Biopsy Collection

Henna markers were applied to the designated treatment areas to ensure precise localization during follow-up examinations and biopsy collection.

After standardized skin cleansing and preparation, a baseline full-thickness skin biopsy (B0) was obtained from a separate pretreatment area using a 4 mm punch. Laser irradiation was then performed within the premarked regions using the assigned energy and spacing parameters. Subsequent full-thickness biopsies were collected from the treated area immediately after treatment (B1) and on Days 3 (B2) and 10 (B3) postprocedure, with a minimum spacing of >10 mm between biopsy sites.

Biopsy sites were protected with sterile adhesive dressings, whereas adjacent observation areas were left uncovered to minimize external influences on the healing process. Standardized clinical photography was performed at baseline and at each follow-up time point. If visible treatment-related skin changes persisted beyond Day 10, photographic follow-up was continued until complete resolution. A schematic representation of the biopsy collection plan and a representative photograph of the henna-marked treatment area are shown in Figure 1.

**Figure 1.**
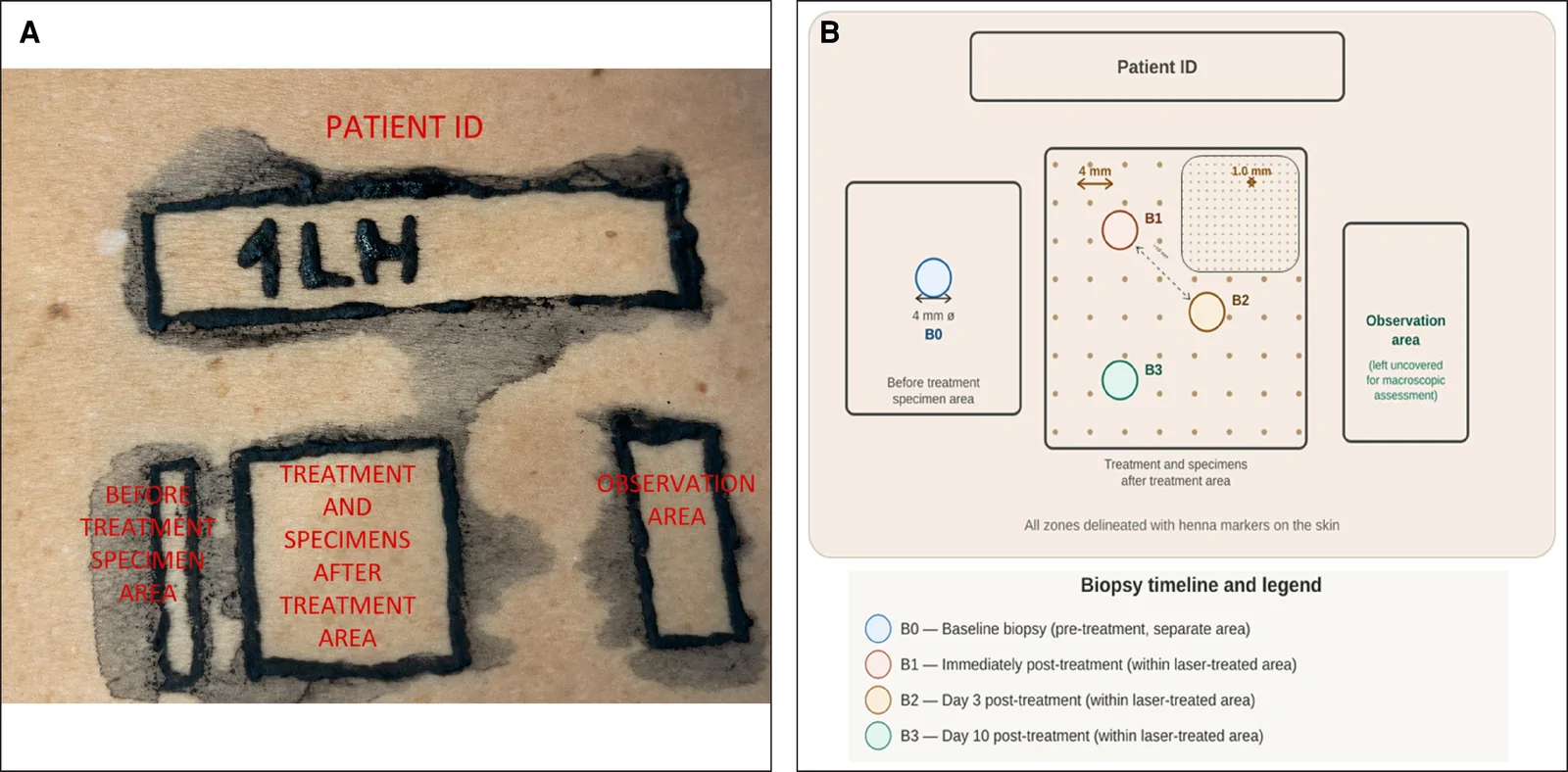
Treatment grid and biopsy collection scheme. (A) Representative clinical photograph of henna-marked skin areas before treatment, illustrating delineation of the patient ID, baseline biopsy zone, treatment and posttreatment biopsy zone, and observation area. (B) Schematic representation of the treatment grid and biopsy sampling plan (not to scale). Three distinct zones were defined: (1) a baseline specimen area (B0) for pretreatment biopsy obtained from a separate, untreated site; (2) a treatment and posttreatment specimen area where laser exposure was applied and sequential biopsies were collected; and (3) an observation area left untreated for macroscopic evaluation. Within the treated area, biopsy sites (B1, B2, and B3) were premarked and spatially separated using a standardized >10 mm grid to minimize overlap of local inflammatory responses. Biopsies were obtained using a 4 mm punch at defined time points: immediately posttreatment (B1), Day 3 (B2), and Day 10 (B3).

### Macroscopic Assessment

Macroscopic evaluation of posttreatment healing was performed by the investigator based on clinical examination and standardized clinical photography, supplemented by participant self-observation. The duration and intensity of erythema, edema, and visible skin reactions were recorded using visual assessment guidelines adapted from the International Contact Dermatitis Research Group (ICDRG) criteria. Complete healing time was defined as the interval from laser treatment to the complete disappearance of all macroscopically visible signs of tissue response, including sub/intraepidermal damage points and any associated erythema or irritation, as determined by investigator assessment. If visible treatment-related changes persisted beyond Day 10, photographic follow-up was continued until complete resolution.

### Microscopic Assessment

Full-thickness skin biopsies were obtained using a 4 mm punch and fixed in 10% neutral buffered formalin. Paraffin-embedded tissue sections were prepared according to standard histological procedures.

Sections were stained with hematoxylin and eosin (H&E) for evaluation of epidermal and dermal morphology, cellular integrity, and inflammatory response. Masson's trichrome staining (Special Stain Kit; DiaPath, Martinengo, Italy) was used to visualize collagen fibers and assess extracellular matrix organization.

Microscopic evaluation was conducted using an ECLIPSE E400 light microscope (Nikon) equipped with a 20× objective. Quantitative analysis included measurements of the height, width, and depth of thermally affected tissue zones to enable correlation between histological findings and applied laser parameters.

## RESULTS

### Macroscopic Evaluation

Complete healing time was defined as the interval from laser treatment to the complete disappearance of all macroscopically visible signs of tissue response, as assessed by the investigator through standardized clinical photography and supported by participant self-observation using visual assessment guidelines adapted from the ICDRG criteria. The macroscopic healing process was characterized by the presence of discrete intraepidermal and subepidermal microdamage points, which gradually exfoliated over time. These changes resolved completely without leaving visible marks on the skin surface.

In the group treated with 28 mJ/point, the mean healing time was 19.7 ± 4.7 days for 4.0 mm spacing (individual values: 16, 18, and 25 days) and 23.0 ± 2.6 days for 1.0 mm spacing (individual values: 20, 24, and 25 days). Transient erythema and mild skin irritation were infrequently observed and occurred primarily in the subgroup treated with dense laser point spacing (1.0 mm). No persistent skin reactions were recorded. In the group treated with 50 mJ/point, the mean healing time was 23.0 ± 3.6 days for 4.0 mm spacing (individual values: 27, 22, and 20 days) and 25.0 ± 2.0 days for 1.0 mm spacing (individual values: 27, 25, and 23 days). In addition to exfoliating microlesions, mild-to-moderate erythema and transient skin irritation were observed within the treated areas, with greater intensity noted in the dense spacing subgroup.

Across all treatment groups, the healing process culminated in complete resolution of visible changes. No bleeding, exudation, postinflammatory hyperpigmentation, or other adverse skin reactions were observed at any stage of follow-up.

### Microscopic Observations of Direct Tissue Interaction

Histological evaluation using H&E and Masson's trichrome staining revealed discrete, vertically oriented columns of tissue alteration in all examined samples. These structures were consistently identifiable at 20× magnification and were confined to localized areas of laser–tissue interaction. Adjacent epidermal and dermal tissue preserved normal histological architecture at both tested energy settings (28 and 50 mJ/point).

Laser spot size and source power were constant across all experiments; variation in delivered energy resulted exclusively from differences in exposure time (16.8 ms for 28 mJ/point and 30 ms for 50 mJ/point).

### Immediate Posttreatment Findings

Immediately after treatment at 28 mJ/point (16.8 ms), a localized intraepidermal blister containing protein-rich fluid was observed at the dermal–epidermal junction. In the combined analysis, the lesion demonstrated a mean height of 113.24 ± 57.75 µm, mean width of 373.83 ± 67.86 µm, and mean basal depth of 184.06 ± 61.27 µm from the outermost stratum corneum surface. The stratum corneum, granular layer, and most of the spinous layer remained intact.

At 50 mJ/point (30 ms), a comparable histological pattern was observed. In the combined analysis, the blister demonstrated a mean height of 111.08 ± 53.50 µm, mean width of 377.01 ± 70.21 µm, and mean basal depth of 179.22 ± 50.78 µm. Thus, in both exposure settings, the superficial epidermal interaction zone showed similar dimensions.

In contrast, the depth of dermal tissue interaction differed between energy settings. Fractional thermal columns visualized by Masson's trichrome staining and assessed in the 1.0 mm spacing groups only reached a mean depth of 350.25 ± 44.00 µm at 28 mJ/point and 426.93 ± 141.73 µm at 50 mJ/point. Quantitative measurements are summarized in Table 2.

**Table 2.** Mean Dimensions of Tissue Interaction Zones Immediately After the Procedure

Parameter (µm)	4.0 mm spacing (*n* = 3)	1.0 mm spacing (*n* = 3)	Combined (*n* = 6)
28 mJ/point (exposure time 16.8 ms)			
Height	115.74 ± 58.48	110.73 ± 62.48	113.24 ± 57.75
Width	373.98 ± 30.21	373.68 ± 96.01	373.83 ± 67.86
Basal depth^a^	183.16 ± 74.02	184.97 ± 52.72	184.06 ± 61.27
Column depth^b^	—	350.25 ± 44.00	350.25 ± 44.00
50 mJ/point (exposure time 30 ms)			
Height	110.68 ± 55.59	111.48 ± 56.62	111.08 ± 53.50
Width	394.85 ± 30.92	359.17 ± 95.53	377.01 ± 70.21
Basal depth^a^	164.09 ± 63.44	194.34 ± 33.17	179.22 ± 50.78
Column depth^b^	—	426.93 ± 141.73	426.93 ± 141.73

All values in µm, expressed as mean ± standard deviation.

^a^Distance from outermost stratum corneum cells to blister base (hematoxylin and eosin staining).

^b^Depth of fractional thermal columns (Masson's trichrome). Measured in 1.0 mm spacing groups only; deep tissue analysis was not performed for 4.0 mm groups.

### Findings at 3 and 10 Days Posttreatment

At 3 days posttreatment, complete reconstitution of the basement membrane and restoration of the spinous and granular layers were observed at both energy settings. A compact residual structure composed of tissue debris covered by the original stratum corneum remained present, with combined mean dimensions of 91.37 ± 28.20 × 359.59 ± 45.96 µm at 28 mJ/point and 111.39 ± 37.76 × 409.16 ± 108.23 µm at 50 mJ/point. In the 1.0 mm spacing groups, the mean residual column depth was 340.08 ± 42.50 µm at 28 mJ/point and 431.26 ± 112.87 µm at 50 mJ/point (Table 3).

**Table 3.** Mean Dimensions of Residual Tissue Interaction Zones 3 Days after the Procedure

Parameter (µm)	4.0 mm spacing (*n* = 3)	1.0 mm spacing (*n* = 3)	Combined (*n* = 6)
28 mJ/point (exposure time 16.8 ms)			
Height	103.19 ± 33.32	79.55 ± 17.41	91.37 ± 28.20
Width	384.13 ± 44.81	335.05 ± 34.56	359.59 ± 45.96
Column depth^a^	—	340.08 ± 42.50	340.08 ± 42.50
50 mJ/point (exposure time 30 ms)			
Height	117.54 ± 16.48	105.23 ± 52.68	111.39 ± 37.76
Width	419.63 ± 80.84	398.69 ± 137.75	409.16 ± 108.23
Column depth^a^	—	431.26 ± 112.87	431.26 ± 112.87

All values in µm, expressed as mean ± standard deviation.

^a^Fractional thermal column depth (Masson's trichrome). Measured in 1.0 mm spacing groups only.

At 10 days posttreatment, all epidermal layers were fully regenerated. A progressively exfoliating residual lesion structure was identified within the stratum corneum, measuring 133.67 ± 34.22 × 390.03 ± 88.35 µm at 28 mJ/point and 127.06 ± 44.47 × 352.49 ± 101.48 µm at 50 mJ/point in the combined analysis. In the 1.0 mm spacing groups, the mean residual column depth was 382.74 ± 89.20 µm at 28 mJ/point and 357.29 ± 75.67 µm at 50 mJ/point (Table 4). At this time point, H&E staining revealed a mild-to-moderate inflammatory infiltrate surrounding areas of altered collagen staining in the dermis beneath the primary interaction zone. Representative macroscopic observation area photographs (Figure 2A) together with the corresponding histological images illustrating the microscopic findings (Figure 2B) for Group V (28 mJ/point, 1.0 mm spacing) are presented in Figure 2. Additional histological and macroscopic documentation from the remaining analyzed groups (Groups II, III, and VI) is provided in Supplemental Figures S1-S3.

**Figure 2.**
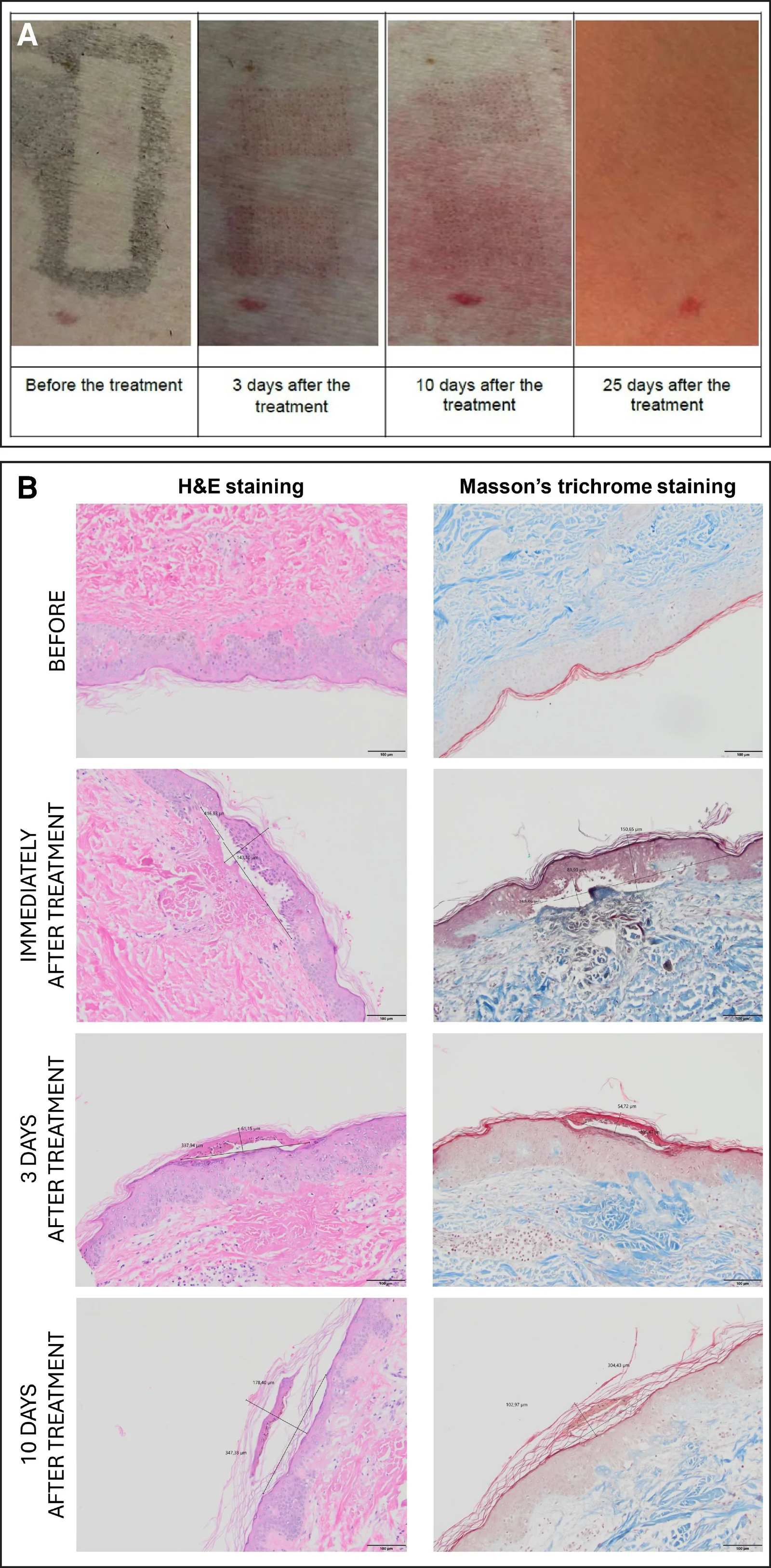
(A) Macroscopic photographs of the observation area documenting the healing course in a representative participant (Case 3ML, Group V: 28 mJ/point, 1.0 mm spacing; a 40-year-old male, back skin). Images taken before treatment, at 3 and 10 days posttreatment, and at complete healing (25 days). (B) Representative histological sections from Group V (28 mJ/point, 1.0 mm spacing) showing tissue interaction and recovery at baseline, immediately after treatment, and at 3 and 10 days posttreatment. Hematoxylin and eosin staining (left) and Masson’s trichrome staining (right) at 20× magnification.

**Table 4.** Mean Dimensions of Residual Tissue Interaction Zones 10 Days after the Procedure

Parameter (µm)	4.0 mm spacing (*n* = 3)	1.0 mm spacing (*n* = 3)	Combined (*n* = 6)
28 mJ/point (exposure time 16.8 ms)			
Height	116.61 ± 31.60	150.73 ± 29.65	133.67 ± 34.22
Width	414.83 ± 101.36	365.22 ± 73.63	390.03 ± 88.35
Column depth^a^	—	382.74 ± 89.20	382.74 ± 89.20
50 mJ/point (exposure time 30 ms)			
Height	117.73 ± 47.47	136.39 ± 43.46	127.06 ± 44.47
Width	320.75 ± 82.00	384.22 ± 116.25	352.49 ± 101.48
Column depth^a^	—	357.29 ± 75.67	357.29 ± 75.67

All values in µm, expressed as mean ± standard deviation.

^a^Fractional thermal column depth (Masson's trichrome). Measured in 1.0 mm spacing groups only.

## DISCUSSION

The present preliminary study provides in vivo histological evidence that fractional nonablative laser treatment using a 1470 nm diode system induces a spatially confined, reproducible, and biologically controlled skin response with morphological features consistent with early tissue remodeling following photothermal stimulation. Across both tested energy settings, the tissue response followed a predictable and physiological healing trajectory, without evidence of uncontrolled epidermal disruption or diffuse dermal damage, suggesting a favorable short-term histologic tolerability of this wavelength and delivery modality. Importantly, this exploratory study was designed to characterize early histological responses rather than to assess clinical efficacy or long-term outcomes.

Fractional laser exposure at both 28 and 50 mJ/point resulted in regularly distributed micro-injury sites corresponding to the predefined pulse grid. Macroscopically, these sites manifested as transient subepidermal dark dots that underwent gradual exfoliation and resolved completely without scarring or pigmentary sequelae. Similar self-limited posttreatment reactions, including mild erythema and transient irritation, have been described for fractional nonablative laser systems and are generally interpreted as manifestations of a controlled inflammatory response rather than pathological tissue injury.^3,5^

Histological analysis confirmed that laser–tissue interaction was primarily localized to the dermal–epidermal junction and the superficial dermis. Immediately after treatment, focal microvesicular alterations were observed at the level of the basement membrane, whereas the stratum corneum and the majority of viable epidermal layers remained structurally preserved. Importantly, no evidence of full-thickness epidermal necrosis, hemorrhage, or widespread tissue disruption was identified. Comparable histological patterns, characterized by epidermal preservation with limited separation at the dermal–epidermal interface, have been reported following fractional nonablative photothermal exposure and are considered characteristic of reversible, nondestructive laser–tissue interaction.^17,18^

Beneath the epidermal interaction zone, localized, vertically oriented zones of collagen condensation were consistently observed within the dermis. The depth of these thermally affected regions increased in proportion to delivered energy, with greater penetration observed at 50 mJ/point compared with 28 mJ/point. This observation is consistent with established biophysical models of laser–tissue interaction, in which prolonged exposure times promote deeper thermal diffusion while maintaining a relatively stable superficial interaction zone.^4,19^ Notably, increasing energy did not result in expansion of superficial epidermal damage, suggesting that once a threshold interaction at the dermal–epidermal junction is achieved, additional energy is preferentially transmitted to deeper dermal compartments rather than laterally within the epidermis.

The temporal evolution of the histological findings suggests a controlled tissue response rather than extensive tissue destruction. Reconstitution of epidermal continuity, including restoration of the spinous and granular layers, was observed by Day 3 posttreatment. By Day 10, reestablishment of the epidermal layers was evident, accompanied by a reduction in the extent of dermal thermal alteration zones and the presence of a mild inflammatory infiltrate, which may reflect ongoing tissue repair processes. At the magnification used in the present analysis (20×), the thermally affected regions were visualized as localized areas of collagen condensation rather than sharply demarcated columns, consistent with early-stage photothermal tissue interaction and subsequent reparative changes.^4,15,17^ Although these morphological findings are compatible with remodeling processes, direct evidence of neocollagenesis cannot be inferred without immunohistochemical or molecular analyses.

Notably, the macroscopic complete healing time of 19.7 to 25.0 days exceeded the histological evidence of epidermal recovery observed at Day 10. This apparent discrepancy likely reflects the distinction between microscopic tissue restoration and the complete resolution of visible surface changes. Although histological analysis at Day 10 confirmed restoration of epidermal continuity and reconstitution of the basement membrane, a compact residual structure composed of tissue debris covered by the original stratum corneum remained present within the superficial epidermis and continued to exfoliate over the following days. This gradually exfoliating debris, manifesting macroscopically as discrete dark dots, represented the dominant visible feature of the posttreatment course and required additional time for complete resolution. The persistence of these macroscopic signs beyond microscopic epidermal recovery is consistent with the normal exfoliation kinetics of the stratum corneum.

An additional observation of potential interest concerns the temporal evolution of fractional thermal column depth. At 28 mJ/point, mean column depth measured 350.25 µm immediately posttreatment, 340.08 µm at Day 3, and 382.74 µm at Day 10. At 50 mJ/point, the corresponding values were 426.93, 431.26, and 357.29 µm, respectively. Although these findings should be interpreted cautiously because of the limited sample size, the initially greater column depth observed at 50 mJ/point was less pronounced by Day 10. This pattern may reflect time-dependent changes in the morphology of the residual thermal interaction zones rather than a persistent difference in tissue response depth.

The present histological observations complement and extend previously published clinical data on 1470 nm nonablative laser therapy. Previous prospective studies have demonstrated significant improvements in acne scars and striae distensae following treatment with this wavelength, with high patient satisfaction and minimal downtime.^10-12^ In those investigations, clinical improvements were documented using objective imaging and biomechanical assessments. The current study provides complementary histological data on early tissue responses following fractional 1470 nm exposure, characterized by localized dermal photothermal alteration and collagen condensation with subsequent epidermal recovery, which is consistent with a controlled, repair-oriented healing pattern. Although the present findings provide important insights into the short-term histologic tolerability and early tissue response profile of fractional 1470 nm laser exposure, clinical effectiveness and long-term therapeutic outcomes require evaluation in appropriately designed outcome-oriented studies.

Notably, the present findings are consistent with recent in vivo histological observations reported for subdermal 1470 nm diode laser systems. In a pilot study by Bollero et al, subdermal delivery of 1470 nm laser energy using a fiber-based system was associated with increased dermal thickness, collagen reorganization, and elastin remodeling at moderate energy levels, with evidence of a nonlinear dose–response relationship. Although that study focused on deep dermal and subcutaneous energy delivery with long-term follow-up, the present investigation complements these observations by characterizing early histological tissue responses following transcutaneous, fractional 1470 nm laser exposure.^20^ Taken together, these findings support the notion that the 1470 nm wavelength can induce controlled dermal tissue responses across different delivery approaches, provided that appropriate energy parameters are applied.

From a biophysical perspective, the observed tissue response reflects the optical and thermal characteristics of the 1470 nm wavelength, which exhibits strong absorption by water and moderate penetration depth. This profile enables efficient conversion of laser energy into localized dermal heat while limiting excessive thermal spread to the epidermis.^8,21^ Such a balance likely contributes to the reproducibility of dermal interaction depth and the favorable safety margin observed across different energy settings.

Several limitations of this study warrant consideration. The sample size is small (*n* = 3 per group), and findings should be interpreted descriptively rather than as generalizable conclusions. No inferential statistical analysis was performed because of the exploratory nature and limited cohort. Histological assessment was not blinded; given the small sample size and the clearly identifiable nature of thermal lesions, blinding was not feasible in this pilot design. Fitzpatrick skin phototypes were not formally documented, limiting the ability to assess phototype-dependent responses. The absence of immunohistochemical or molecular markers (eg, collagen types I and III [COL I/III], Ki-67 proliferation marker [Ki-67], heat shock protein 47 [HSP47], and transforming growth factor beta [TGF-β]) precludes definitive conclusions regarding neocollagenesis or true dermal remodeling. The histological follow-up was limited to 10 days, which is insufficient to assess longer-term regenerative effects, pigmentary sequelae, or delayed adverse outcomes; macroscopic follow-up extended to 27 days but did not include standardized adverse event grading. Comprehensive safety assessment would require larger cohorts, documentation of Fitzpatrick phototypes, a standardized AE scale, and extended follow-up of at least 4 to 12 weeks. The study evaluated a single device platform, which may limit the generalizability of findings to other 1470 nm laser systems. Despite these limitations, the high reproducibility of histological patterns across participants and their concordance with macroscopic observations support the descriptive validity of these preliminary findings. Future studies should address these limitations with larger cohorts, blinded histopathological assessment, extended follow-up intervals, immunohistochemical confirmation of remodeling, and standardized safety endpoints.

## CONCLUSIONS

The findings of this study indicate that fractional exposure to 1470 nm laser energy elicits a predictable, time-dependent tissue response characterized by rapid restoration of epidermal continuity and localized dermal thermal effects, including areas of collagen condensation. Within the short-term observation period, the healing pattern was consistent across samples and proceeded without disruption of overall skin integrity or evidence of clinically relevant adverse tissue reactions. These observations suggest that the 1470 nm wavelength may offer a favorable balance between optical penetration and water absorption, allowing selective photothermal interaction within the dermis while preserving the epidermis. Within the limitations of this exploratory in vivo study, the LaserMe system demonstrates a favorable short-term histologic tolerability profile; these findings cannot be extrapolated to conclusions regarding clinical safety, which requires longer follow-up and larger cohorts.

## Supplementary Material

ojag161_Supplementary_Data

## References

[1] Anderson RR, Parrish JA. Selective photothermolysis: precise microsurgery by selective absorption of pulsed radiation. Science. 1983;220:524–527. doi: 10.1126/science.6836297 6836297

[2] Massey RA, Marrero G, Goel-Bansal M, Gmyrek R, Katz BE. Lasers in dermatology: a review. Cutis. 2001;67:477–484. 11419019

[3] Alexiades-Armenakas MR, Dover JS, Arndt KA. The spectrum of laser skin resurfacing: nonablative, fractional, and ablative laser resurfacing. J Am Acad Dermatol. 2008;58:719–737. doi: 10.1016/j.jaad.2008.01.003 18423256

[4] Manstein D, Herron GS, Sink RK, Tanner H, Anderson RR. Fractional photothermolysis: a new concept for cutaneous remodeling using microscopic patterns of thermal injury. Lasers Surg Med. 2004;34:426–438. doi: 10.1002/lsm.20048 15216537

[5] Fisher GH, Geronemus RG. Short-term side effects of fractional photothermolysis. Dermatol Surg. 2005;31:1245–1249. doi: 10.1111/j.1524-4725.2005.31934 16176779

[6] Tannous Z . Fractional resurfacing. Clin Dermatol. 2007;25:480–486. doi: 10.1016/j.clindermatol.2007.05.007 17870526

[7] Ross EV, Naseef GS, McKinlay JR, et al Comparison of carbon dioxide laser, erbium:YAG laser, dermabrasion, and dermatome: a study of thermal damage, wound contraction, and wound healing in a live pig model: implications for skin resurfacing. J Am Acad Dermatol. 2000;42:92–105. doi: 10.1016/s0190-9622(00)90016-1 10607327

[8] Jacques SL . Optical properties of biological tissues: a review. Phys Med Biol. 2013;58:R37–R61. doi: 10.1088/0031-9155/58/11/R37 23666068

[9] Husain Z, Alster TS. The role of lasers and intense pulsed light technology in dermatology. Clin Cosmet Investig Dermatol. 2016;9:29–40. doi: 10.2147/CCID.S69106 PMC4745852 26893574

[10] Kubik P, Bighetti S, Bettolini L, et al Effectiveness and safety of the use of 1470 nm laser therapy in patients suffering from acne scarring of the facial skin. Clin Cosmet Investig Dermatol. 2025;18:543–551. doi: 10.2147/CCID.S510208 PMC11911818 40099044

[11] Kubik P, Bighetti S, Bettolini L, et al The effectiveness and safety of 1470 nm non-ablative laser therapy for the treatment of striae distensae: a pilot study. Cosmetics. 2025;12:148. doi: 10.3390/cosmetics12040148

[12] Kubik P, Gruszczyński W, Łukasik B. Assessment of safety and mechanisms of action of the 1470 nm LaserMe device. Clin Case Rep Int. 2024;8:1683. doi: 10.25107/2638-4558.1683

[13] dos Santos IFB, Guidi RM, Bulhões VCS, de Rabelo VA. Segurança e efetividade da utilização de laser fracionado não ablativo de 1470 nm em paciente de fototipo v—relato de caso. Aesthetic Orofac Sci. 2025;6:66–71. doi: 10.51670/aos.v6i3.286

[14] Nilforoushzadeh MA, Heidari A, Ghane Y, et al The endo-lift laser (intralesional 1470 nm diode laser) for dermatological aesthetic conditions: a systematic review. Aesthetic Plast Surg. 2024;48:5097–5114. doi: 10.1007/s00266-024-04082-2 38886198

[15] Oh BH, Hwang YJ, Lee YW, Choe YB, Ahn KJ. Skin characteristics after fractional photothermolysis. Ann Dermatol. 2011;23:448–454. doi: 10.5021/ad.2011.23.4.448 PMC3229937 22148011

[16] Lifestyle Aesthetics. LaserMe. Accessed February 10, 2026. https://ls-aesthetics.com/devices/laserme/

[17] Hantash BM, Bedi VP, Kapadia B, et al In vivo histological evaluation of a novel ablative fractional resurfacing device. Lasers Surg Med. 2007;39:96–107. doi: 10.1002/lsm.20468 17311274

[18] Schallen KP, Seder M, Jorgenson M, Schomacker K, Bhawan J. In vivo histological study evaluating non-ablative fractional 1940-nm laser. Lasers Surg Med. 2025;57:54–62. doi: 10.1002/lsm.23842 PMC11776451 39279229

[19] Ansari MA, Erfanzadeh M, Mohajerani E. Mechanisms of laser-tissue interaction: II. Tissue thermal properties. J Lasers Med Sci. 2013;4:99–106. PMC4295363 25606316

[20] Bollero D, Orlando G, Dell’Avanzato R, et al In vivo pilot study of the effects of a subdermal 1470 nm diode laser on human skin. Laser Ther. 2025;32:31–40. doi:10.4081/ltj.2025.422

[21] Welch AJ, van Gemert MJC. Optical-Thermal Response of Laser-Irradiated Tissue. Springer Science & Business Media; 2011.

